# Your teaching strategy matters: how engagement impacts application in health information literacy instruction *

**DOI:** 10.5195/jmla.2017.8

**Published:** 2017-01

**Authors:** Heather A. Johnson, Laura Barrett

## Abstract

**Objective:**

The purpose of this study was to compare two pedagogical methods, active learning and passive instruction, to determine which is more useful in helping students to achieve the learning outcomes in a one-hour research skills instructional session.

**Methods:**

Two groups of high school students attended an instructional session to learn about consumer health resources and strategies to enhance their searching skills. The first group received passive instruction, and the second engaged in active learning. We assessed both groups’ learning using 2 methods with differing complexity. A total of 59 students attended the instructional sessions (passive instruction, n=28; active learning, n=31).

**Results:**

We found that the active learning group scored more favorably in four assessment categories.

**Conclusions:**

Active learning may help students engage with and develop a meaningful understanding of several resources in a single session. Moreover, when using a complex teaching strategy, librarians should be mindful to gauge learning using an equally complex assessment method.

## INTRODUCTION

Active learning is a pedagogical approach in which educators act as facilitators of learning rather than as didactic lecturers, encouraging students to engage meaningfully with educational content [1]. Active learning comprises strategies that “involve students doing things and thinking about the things they are doing” [2], including student collaboration, reflection, exploration, and critical thinking [3–5]. Students in active learning environments become partners in the teaching and learning process, and they interact meaningfully with content and think creatively about information, resulting in deeper learning [5]. This method of learning is considered to be more effective than passive instruction, as it emphasizes teamwork, instills a sense of responsibility in individual group members, and enhances cognition [3, 6].

The effectiveness of active learning has been demonstrated repeatedly, and educators have published widely on the broad applicability of this pedagogical method. Faculty use of active learning methods, particularly collaborative student work followed by faculty explanation, has been shown to improve student learning more than either collaborative work or faculty explanation alone [7]. Numerous degree programs that prepare students for health professions mandate the inclusion of active learning pedagogy, due to its demonstrated effectiveness in developing higher-order thinking skills among students [8]. Linton and colleagues argue for the value of using multiple active learning pedagogies to enhance learning and to achieve other educational objectives, including increased collaboration and development of better verbal communication skills [1].

In the summer of 2015, the authors used two teaching methods, active learning and direct instruction, in two one-shot instructional sessions for high school students participating in one of Dartmouth College’s two weeklong Health Careers Institutes. The goal of the one-shot sessions was to introduce students to health information resources and concepts that were intended to optimize their searching capabilities.

## METHODS

### Student characteristics

Health Careers Institute participants were high school students. A total of 59 students attended 1 of the 2 weeks: 58 were from the United States, and 1 was from Hong Kong. Of the 59 students who participated in the program, 28 attended the passive instruction session, and 31 attended the active learning session. Most (87%) of the participants were female, with only a slight variation between the 2 groups (89% in the passive instruction group, 84% in the active learning group). Across groups, student age ranged from 14–17 years, with an average age of 15.5 years (standard deviation=0.88).

### Learning objectives

By the end of the session, students in both groups should have been able to: (1) identify and use three consumer health resources to find reliable health information, (2) apply appropriate strategies to optimize searches, (3) appraise sources for authority and potential bias, and (4) properly cite sources.

### Instruction sessions

#### Passive instruction session

In the passive instruction session, we used a didactic lecture to introduce the students to three consumer health resources: MedlinePlus, PubMed Health, and the patient information contained in UpToDate. (Although we introduced students only to patient information, we refer to this source simply as UpToDate from this point forward.) In addition to these resources, we also emphasized the importance of using precise vocabulary (e.g., “mononucleosis” versus “mono”) to optimize Google searches and demonstrated how to utilize Google’s Advanced Search feature. We also discussed Wikipedia and engaged students in a short discussion about its uses and limitations.

#### Active learning session

In the active learning session, we introduced students to content via the Jigsaw Method †. Using this method, we randomly assigned students to one of six groups, with each group containing five or six students. Within these groups, students became content experts about one of six prescribed resources: PubMed Health, UpToDate, MedlinePlus, Google Scholar, Google, and Wikipedia. We then formed new groups including at least one member from each of the original groups, and each student led a short discussion on their assigned areas of expertise. After students learned from one another in their heterogeneous groups, we delivered a short lecture on the importance of source citation and then facilitated a brainstorming activity to further synthesize the information discussed in the heterogeneous groups.

### Assessment

Didactic instruction is a low-complexity teaching technique that is best assessed using a low-complexity assessment strategy [9], such as a “Quiz Bowl.” On the other hand, the Jigsaw Method is a high-complexity strategy requiring an assessment method that measures students’ ability to engage in high-complexity activities. Therefore, we used both low-complexity and high-complexity assessment methods to evaluate student learning.

At the close of each of the two instructional sessions, we utilized a low-complexity *Jeopardy*-style Quiz Bowl to assess students’ learning and to reinforce concepts. Six categories of questions represented each of the six topics, and each category contained five questions. Although a point value was assigned to each question, the difficulty of questions was not correlated with their numerical values.

Also, as part of their participation in the Health Careers Institute, students worked in groups to write a research paper and produce a bibliography that was due at the end of the week. Students in the passive instruction group researched schizophrenia and amyotrophic lateral sclerosis, whereas students in the active learning group researched chikungunya, Lyme disease, tapeworm, and necrotizing fasciitis. We utilized a high-complexity assessment of how well students were able to apply the information by using a rubric to score the quality of students’ bibliographies (supplemental appendix). Bibliographies were stripped of identifying information, so we were not able to tell which group the bibliography originated from. The rubric consisted of six categories: (1) use of MedlinePlus, PubMed Health, and UpToDate patient information; (2) use of commercial sources; (3) use of government and nonprofit sources (other than MedlinePlus and PubMed Health); (4) use of Wikipedia; (5) use of scholarly articles; and (6) accuracy of citations in the bibliographies. The rubric also contained three levels of achievement: exceeds expectations, meets expectations, and does not meet expectations. We received institutional review board approval to review students’ bibliographies and analyze them against this rubric.

## RESULTS

During the Quiz Bowl, the passive instruction group correctly answered every question, whereas the active learning group correctly responded to approximately one-half of the questions. These results suggested that passive instruction was more effective at activating students’ short-term memory.

**Figure 1:**
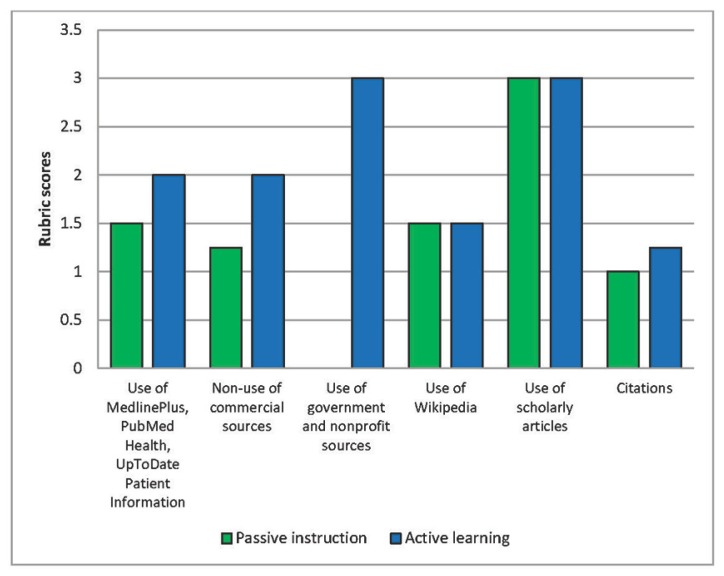
Differences between passive instruction and active learning groups in bibliographic quality

To evaluate students’ ability to apply the information, we evaluated both groups’ bibliographies. The active learning group scored more favorably than the passive instruction group in four areas: use of MedlinePlus, PubMed Health, and UpToDate; use of commercial sources; use of government and nonprofit sources (other than MedlinePlus and PubMed Health); and number of citation errors in the bibliographies. Both groups demonstrated an equal propensity to cite at least three scholarly articles, as well as an equal propensity to cite Wikipedia (Figure 1).

## DISCUSSION

Active learning is a pedagogical approach in which educators facilitate learning and empower students to take ownership of their education by making them responsible for engaging with content [8]. To gain primary experience with this pedagogical approach, we compared the two teaching methods using a *Jeopardy*-style game that tested for rote memorization as well as a rubric that assessed bibliographic quality. When compared with the active learning group, the passive instruction group performed more favorably in terms of their ability to immediately recall information but did not perform as well in terms of their ability to produce a high-quality bibliography. That is, students in the active learning group created bibliographies that included a broader range of reputable sources, demonstrating their ability to effectively search for and evaluate health information.

In line with Van Amburgh and colleagues’ notion that the assessment strategy must match the teaching method in terms of its level of complexity, it is, in hindsight, not surprising that the passive instruction group performed better when they were tested for immediate fact recall and that the active learning group scored higher on their bibliographies [9]. While it was disappointing that the active learning group did not perform as well as the passive instruction group during the Quiz Bowl activity, it is important to remember that rote memorization is less useful than the ability to apply knowledge when doing research.

Our study has limitations. First, we reviewed only six bibliographies: two from the passive instruction group and four from the active learning group, which limits the reliability of our findings. Second, because the passive instruction and active learning groups researched different topics, it is impossible to designate one group as a control and another as experimental. Third, it was impossible to assess whether students used PubMed Health for purposes other than locating general information on a specific topic. When the rubric was designed, a section was dedicated to assessing whether students used the three main consumer health resources (MedlinePlus, PubMed Health, and UpToDate) to which they had been introduced. However, PubMed Health includes a discovery tool that leads searchers to informative content and scholarly articles. Although no students cited PubMed Health, this does not mean that PubMed Health was not used as an interface for discovering articles indexed in MEDLINE.

In the summer of 2016, we will teach all library instructional sections of the Health Careers Institute using only active learning methods. We arrived at this decision based on our previous experience coupled with deeper research into the efficacy of active learning methods. We recommend that librarians incorporate active learning methods into their own teaching in order to promote deep and focused engagement with content, resulting in higher rates of retention. Biomedical librarians should be mindful of their learning objectives and incorporate appropriate pedagogical approaches that best support those objectives.

## SUPPLEMENTAL FILE

Appendix Rubric to score the quality of students’ bibliographies

## Footnotes

† Two books that can help instructors select active learning techniques, including the Jigsaw Method, that support their instructional settings and learning objectives are:

- Barkley EF, Cross KP, Major CH. Collaborative learning techniques: a handbook for college faculty. San Francisco, CA: Jossey-Bass; 2005. 303 p.
- Rutherford P. Active learning and engagement strategies. Alexandria, VA: Just ASK Publications; 2012. 170 p.
