# Supplementary material for: Your teaching strategy matters: how engagement impacts application in health information literacy instruction
Source: J Med Libr Assoc. 2017 Jan;105(1):44–8. doi: 10.5195/jmla.2017.8 (PMC5234460; doi:10.5195/jmla.2017.8)
Supplement: Appendix [file jmla_jan17_johnson_app.pdf]

JMLA Journal of the Medical Library Association

SC

Supplemental content to
J Med Libr Assoc. Jan;105(1): dx.doi.org/10.5195/jmla.2017.8
www.mlanet.org
© Johnson, Barrett 2017

Your teaching strategy matters: how engagement impacts application in health information literacy instruction

Heather A. Johnson, MLIS; Laura C. Barrett, MSI

APPENDIX

Rubric to score the quality of students' bibliographies

	3 (Exceeds expectations)	2 (Meets expectations)	1 (Does not meet expectations)
Sources: MedlinePlus, PubMed Health, UpToDate Patient Information	Student uses all 3 sources to support evidence	Student uses 1 or 2 of the 3 sources to support evidence	Student uses none of the sources to support evidence
Commercial sources		Student does not use any commercial (.com) sources to support claims (excluding Wikipedia and periodicals)	Student uses one or more commercial (.com) sources to support claims (excluding Wikipedia and periodicals)
Government and nonprofit sources	Student uses 1 or more government or nonprofit sources (such as the Centers for Disease Control and Prevention and World Health Organization, excluding MedlinePlus, PubMed Health, and National Center for Biotechnology Information) to support evidence		
Wikipedia		Student does not cite Wikipedia	Student cites Wikipedia
Scholarly articles	Student cites 2 or more scholarly research articles	Student cites 1 scholarly research article	Student does not cite any scholarly research articles
Citations	Bibliography contains no citation errors	Bibliography contains 1–3 citation errors	Bibliography contains more than 3 errors
Maximum number of points: 14<br>Meets expectations: 10<br>Minimum number of points: 5			
